# Integrated Transcriptome and Metabolome Analysis Reveals the Mechanism of Sweetness Formation in Vegetable Soybean Seeds

**DOI:** 10.3390/molecules31091485

**Published:** 2026-04-29

**Authors:** Xiaotian Yuan, Lu Huang, Jinyang Liu, Xiaoyan Zhang, Ziyan Lu, Qingyang Li, Xingxing Yuan, Xin Chen, Chenchen Xue

**Affiliations:** 1 College of Food Science and Engineering, Nanjing University of Finance & Economics, Nanjing 210023, China; yuanxiaotian235@163.com; 2 Institute of Industrial Crops, Jiangsu Academy of Agricultural Sciences, Nanjing 210014, China; 20200024@jaas.ac.cn (J.L.); xyzhang@jaas.ac.cn (X.Z.); yxx@jaas.ac.cn (X.Y.); cx@jaas.ac.cn (X.C.); 3 School of Food and Biological Engineering, Jiangsu University, Zhenjiang 212013, China; luzyyy1@163.com (Z.L.); leeshi1874@163.com (Q.L.)

**Keywords:** vegetable soybean, sweetness, metabolomic, transcriptomic, network analysis

## Abstract

To understand the mechanism of sweetness formation in vegetable soybean seeds, an integrative transcriptomic and metabolomic analysis was conducted using high sweetness (HS) and low sweetness (LS) varieties selected from 287 resources based on electronic tongue evaluation. The HS variety exhibited significantly higher levels of soluble sugars (58.91 mg/g) and free amino acids (54.15 mg/g). Transcriptomic results indicated that DEGs correlated with glycolysis/gluconeogenesis, starch and sucrose metabolism, and biosynthesis of amino acids pathways were significantly up-regulated in the HS variety. Metabolomic analysis showed that DAMs were significantly enriched in the biosynthesis of secondary metabolites, the amino acid biosynthesis, and the pentose phosphate pathway. Co-expression network analysis further demonstrated correlations between DEGs and DAMs related to glycolysis/gluconeogenesis and amino acid biosynthesis. Eight candidate genes related to sweetness formation were identified through transcriptomic data and validated by RT-qPCR. The present findings represent a fundamental advance in understanding the regulatory mechanisms underlying the sweetness of vegetable soybeans.

## 1. Introduction

Soybean (*Glycine max* (L.) Merr.) is widely acknowledged as a globally significant crop [1]. This widely cultivated species is valued for its multiple uses, including grain, oil, and forage production [2]. The classification of soybeans is determined by harvest timing, with two primary categories being grain and vegetable soybeans. Grain soybeans are typically collected upon reaching full maturation (R8 growth stage), with their average contents of protein, oil, carbohydrate and ash generally standing at approximately 40%, 20%, 35%, and 5%, respectively [3]. Owing to their high levels of protein and oil, grain soybeans are widely used in producing human food products and animal feed [4]. Vegetable soybeans (edamame) are usually harvested during the R6-R7 developmental stages [5]. At this growth phase, the pods develop into a plump shape with vivid green hues, and the seeds inside reach full morphological development [6]. These soybeans are popular among consumers primarily due to their tender texture and rich nutritional profile, which includes vitamins, amino acids, minerals, protein, fiber, and health-beneficial isoflavones [7]. Vegetable soybeans are among the few botanical foods that supply amino acids like lysine and tryptophan. In addition, they are abundant in sugars and minerals [8]. Renowned for its flavor and nutritional value, vegetable soybeans have become a globally popular food, with demand steadily increasing.

Vegetable soybeans have three main food quality attributes: sensory quality, appearance quality, and nutritional quality [9,10]. Sensory quality mainly includes sweetness, texture, aroma, and mouthfeel, which significantly influence consumer preference. Sensory evaluations indicate a positive correlation between the sweetness of vegetable soybeans and consumer acceptance [11], with consumers generally favoring sweeter varieties. The sweetness of vegetable soybeans is influenced by soluble sugars, free amino acids, organic acids, and sweet-tasting substances. The key soluble sugars contributing to sweetness are sucrose [12], with sucrose being the dominant factor [13,14,15]. Furthermore, the perception of sweet taste is also affected by several amino acids, namely alanine, threonine, glycine, serine and proline [9,16].

Research on sweetness in vegetable soybean currently focuses mainly on phenotypic investigations, particularly on the accumulation patterns and formation mechanisms of individual chemical components [17,18]. Song et al. [12] used omics analysis to identify traits that contribute to vegetable soybean quality, including various types of sugars. In a more targeted genetic study, Xu et al. [19] identified nine potential genes related to the amount of soluble sugar in vegetable soybean. The mechanisms underlying sweetness or flavor formation have previously been documented in various other plant species. Zhou et al. [20] performed a study for analyzing the transcriptomes and metabolomes of different chromosome numbers cucumber fruits at varying stages of development. The gene *Csa_5G322500* was identified as a regulator of soluble sugar levels in cucumber fruits. Gou et al. [21] performed metabolomic and transcriptomic analyses on three different apricot varieties at three developmental stages. Their findings revealed that VIP transcription factors, sugar transport proteins, along with their corresponding lncRNAs and miRNAs, act as core regulators in the transcriptional control of sugar accumulation. Min et al. [22] investigated the molecular basis of flavor and sweetness variations between postharvest and vine-ripened tomato fruits using an integrated transcriptomic and metabolomic approach. Their study identified seven hub genes critical for the control of fruit flavor. Nie et al. [23] employed a two-omics strategy in order to elucidate the gene networks that regulate sugar metabolism in plum. The research identified 4 principal sugars.

However, limited research has focused on the genomic basis of sweetness development in vegetable soybeans. In this study, 287 vegetable soybean accessions from diverse regions across China were collected. Seed sweetness was quantified using an electronic tongue, after which high and low sweetness varieties were selected for combined metabolomic and transcriptomic analyses. Through integrated omics analysis, differential metabolites and differentially expressed genes associated with seed sweetness were identified, and key metabolic pathways contributing to sweet taste formation were further elucidated. These findings help clarify the molecular mechanism of sweet taste quality in vegetable soybeans. Moreover, this study addresses industrial development needs and market demand, offering practical insights for the utilization and development of high-quality, high-sweetness vegetable soybean resources.

## 2. Results

### 2.1. Identification of Soluble Sugar, Organic Acid and Free Amino Acids Components in Vegetable Soybean

**Figure 1:**
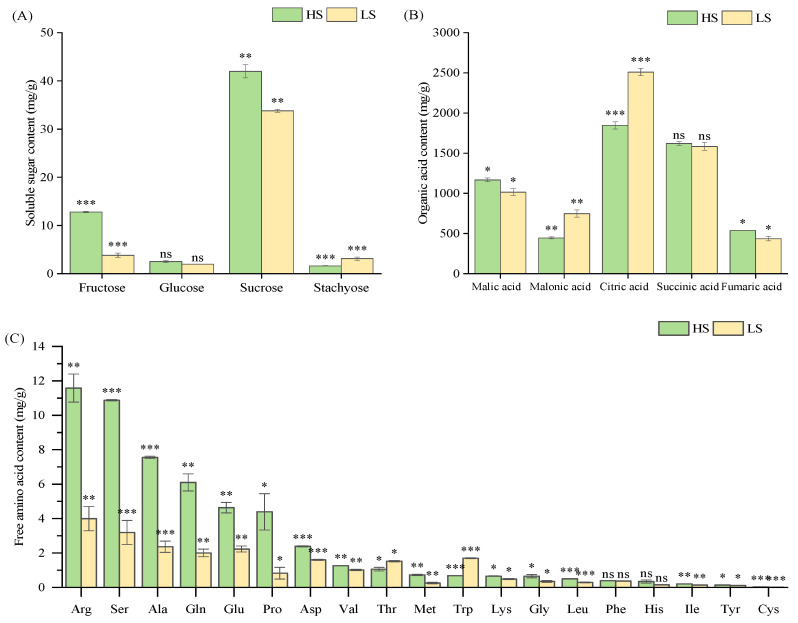
Chemical composition analysis in high-sweetness (HS) and low-sweetness (LS) vegetable soybeans. (**A**) Soluble sugar content. (**B**) Organic acid content. (**C**) Free amino acid fraction. Data are presented as mean ± SD (*n* = 3 biological replicates per group). *, **, *** indicate significant difference at *p* < 0.05, *p* < 0.01 and *p* < 0.001, respectively. ns indicates not significant (*p* ≥ 0.05).

The sugar composition of vegetable soybean varieties was analyzed, including fructose, glucose, sucrose, raffinose, and stachyose content. Experimental materials consisted of high-sweetness (HS) and low-sweetness (LS) varieties. Compared to the LS variety, the HS variety accumulated significantly more sucrose, fructose and glucose (*p* < 0.05; Figure 1A), a result that aligned with the electronic tongue data. Raffinose was not detected in any varieties. Sucrose was the predominant sugar, with concentrations of 41.99 mg/g in the HS variety versus 33.79 mg/g in the LS variety. Fructose levels ranked second, showing a 3.3-fold higher concentration in HS (12.78 mg/g) than in the LS variety (3.82 mg/g). Glucose content, although relatively low overall, remained significantly higher in HS (2.53 mg/g) than in the LS variety (1.97 mg/g). The total soluble sugar content was 58.91 mg/g in the HS variety and 42.69 mg/g in the LS variety. In contrast, stachyose exhibited an inverse pattern, with the LS variety containing nearly twice the amount (3.12 mg/g) found in the HS variety (1.62 mg/g).

The organic acid profiles differed significantly between the two varieties. Organic acids are not only important intermediates in the tricarboxylic acid cycle, participating in metabolic processes such as respiration and photosynthesis, but also in amino acid biosynthesis and cell pH regulation [24]. In this study, quantification was performed on several key organic acids, including malonic, citric, succinic, malic, and fumaric acid. Among the five organic acids analyzed, citric acid showed the highest concentration, with levels of 1845.46 μg/g in the HS variety and 2509.35 μg/g in the LS variety (Figure 1B). Notably, citric acid has been reported to enhance cellular perception of sucrose sweetness [25]. Succinic acid concentrations did not differ significantly between varieties. Regarding sweetness perception, sucrose sweetness is generally the least suppressed quality while simultaneously acting as the strongest suppressor of other tastes [26]. In addition, the sweetness of fruits and vegetables is determined not only by the types and concentrations of sugars present, but also by their organic acid composition. The perceived intensity of sweetness in the overall flavor profile is governed by the sugar-to-acid ratio [27]. Compared to the LS variety, the HS variety had significantly elevated sugar levels (especially sucrose) and reduced organic acid levels, which is the probable cause of its higher sweetness intensity.

Nineteen free amino acids were detected in both HS and LS varieties, with asparagine (Asn) being the sole undetected compound (Figure 1C). Total free amino acid content was 2.4-fold higher in the HS variety (54.15 mg/g) than in the LS variety (22.40 mg/g). Notably, levels of key sweet amino acids, including serine (Ser), alanine (Ala), glycine (Gly), and proline (Pro), were substantially higher in the HS variety.

### 2.2. Transcriptome Analysis

**Figure 2:**
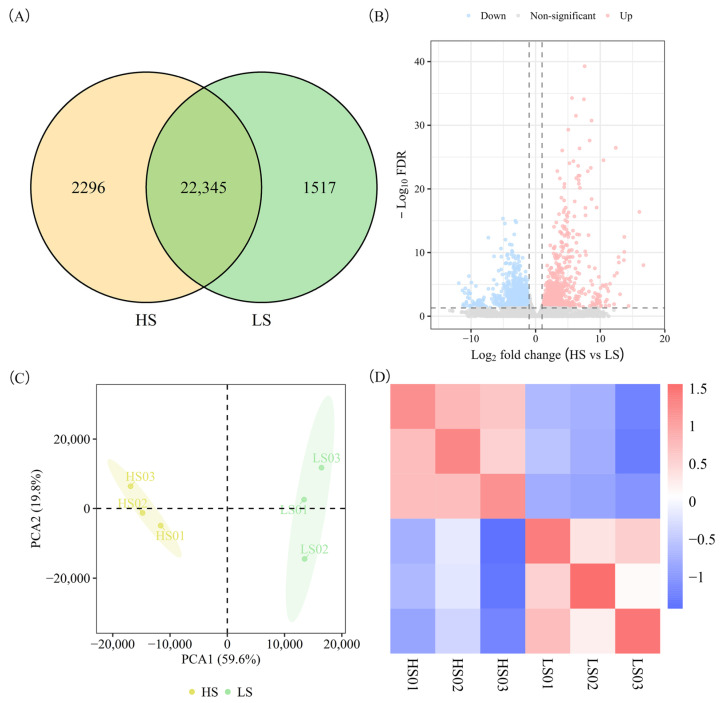
Overview of the transcriptome analysis in HS and LS vegetable soybean varieties. (**A**) Venn diagram of DEGs from HS and LS samples. (**B**) Volcanic maps of gene expression in HS and LS samples. Vertical dashed lines: |log2FC| = 1; horizontal dashed line: FDR = 0.05. (**C**) Principal component analysis of gene expression profiles. (**D**) Correlation analysis between HS and LS samples.

The sequencing analysis detected 26,158 expressed genes, with 2296 being HS-specific and 1517 LS-specific (Figure 2A). The comparison of the HS and LS varieties revealed 2897 DEGs, with 1942 upregulated and 955 downregulated. (Figure 2B). PCA of the transcriptome data showed a clear separation between HS and LS varieties in the scatter plot (Figure 2C), with PC1 and PC2 explaining 59.6% and 19.8% of the total variance, respectively. Biological replicates showed high consistency (Figure 2D), which confirms the reliability of our dataset. Collectively, transcriptomic analyses demonstrate substantial differentiation between the HS and LS varieties at the gene expression level.

Gene ontology (GO) terms are classified into three domains: molecular function (MF), cellular component (CC), and biological process (BP). Approximately 50% of the identified DEGs were enriched in the BP domain (Figure S2). In the MF ontology of GO terms, catalytic activity and binding exhibited significant enrichment. Within the CC group, enrichment of genes in cell and cell part represented the predominant pathways. KEGG analysis demonstrated that all DEGs were assigned to 124 pathways. These pathways belonged to five primary KEGG categories. Among these, the Metabolism category encompassed the highest number of both pathways and associated DEGs. The most significantly enriched KEGG terms included: starch and sucrose metabolism, carbon metabolism, and biosynthesis of amino acids. Furthermore, DEGs were significantly enriched in glycolysis/gluconeogenesis, photosynthesis, and pentose phosphate. All these enriched KEGG pathways fall under the primary “Metabolism” category and showed significant enrichment (*p* < 0.05). Notably, within the primary category “Metabolism”, “metabolic pathways” was the most representative and contained the highest number of DEGs. Compared to the low-sweetness variety, DEGs of the high-sweetness variety showed predominant upregulation. In contrast, DEGs associated with the “biosynthesis of secondary metabolites” pathway exhibited predominant downregulation, revealing distinct metabolic divergence between the two groups.

### 2.3. Differentially Expressed Genes Related to Seed Sweetness

**Figure 3:**
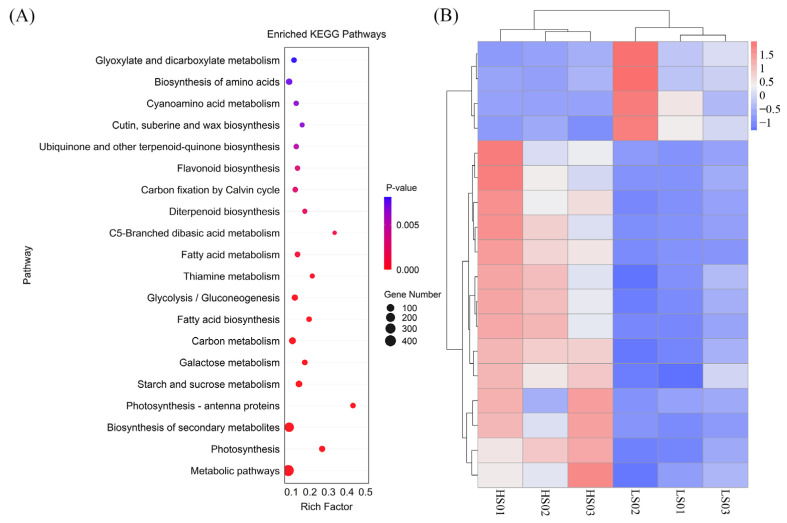
Expression profiles of DEGs in HS and LS vegetable soybean varieties. (**A**) Bubble map of the top 20 KEGG pathways enriched with DEGs from different vegetable soybean samples. (**B**) Heatmap of expression levels for sweetness-related genes in different vegetable soybean varieties.

A comprehensive analysis of the KEGG database revealed three pivotal pathways that are closely associated with the perception of seed sweetness. (Figure 3A). Most DEGs of high-sweetness variety in these pathways showed upregulation patterns. Specifically, 78% were upregulated in glycolysis/gluconeogenesis, 72% in starch and sucrose metabolism, and 82% in amino acid biosynthesis. Only a small proportion of genes (18~28% across pathways) exhibited downregulation. Through screening, a total of 17 DEGs were identified (Figure 3B). Within glycolysis/gluconeogenesis, 13 DEGs were identified, including: 1 hexokinases (*HXK2*, *Glyma.01G007300*), 1 phosphofructokinase (*PFK3*, *Glyma.08G199800*), 2 fructose-1,6-bisphosphatases (*FBP*, *Glyma.07G142700*, *Glyma.18G19360*), 3 fructose-bisphosphate aldolases (*FBA2*, *Glyma.11G111100*, *Glyma.11G111400*, *Glyma.12G037400*), 1 glucose-6-phosphate isomerase 1 (*PGI1*, *Glyma.06G094300*), 1 phosphoenolpyruvate carboxykinase (*PCKA*, *Glyma.06G091500*), 2 pyruvate kinases (*PKP2*, *Glyma.10G227800*, *Glyma.16G173100*), 1 phosphoglycerate kinase (*PGK1*, *Glyma.15G261900*) and 1 glyceraldehyde-3-phosphate dehydrogenase (*GAPCP1*, *Glyma.03G092700*). Within the “starch and sucrose metabolism“ pathway, 3 DEGs were screened, 1 invertase (*INV*, *Glyma.12G005100*), 1 *HXK2*, and 1 *PGI1*. Among them, *HXK2* and *PGI1* are included in the “glycolysis/gluconeogenesis” pathway. Within the “biosynthesis of amino acids” pathway, 2 DEGs were screened, including: 1 aspartate kinase (*AK1*, *Glyma.16G049300*) and 1 threonine synthase (*TS1*, *Glyma.17G052000*). In addition, a stachyose synthase (*STS1*, *Glyma.19G217700*) has also been screened.

### 2.4. Metabolome Analysis

**Figure 4:**
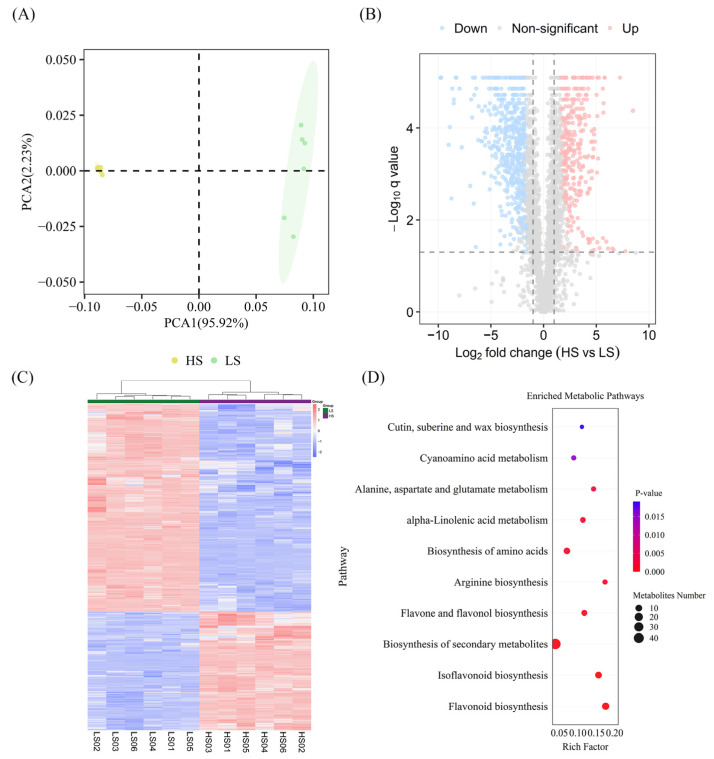
Metabolic characteristics of HS and LS vegetable soybean varieties. (**A**) Principal component analysis diagram of metabolites. (**B**) Volcanic maps of DAMs across samples. Vertical dashed lines: |log2FC| = 1; horizontal dashed line: q-value = 0.05. (**C**) Heatmap of DAM expression levels in different samples. (**D**) Bubble plot of KEGG pathways enriched with DAMs.

PCA demonstrated high intra-group reproducibility among biological replicates (Figure 4A). The initial PC1 and PC2 elucidated 95.92% and 2.23% of the aggregate variance, collectively constituting 98.15% in sum. The PCA score plot revealed distinct clustering of the HS and LS groups, further confirming significant metabolic differences between them.

An OPLS-DA model was applied to further screen for differentially accumulated metabolites (DAMs) (Figure S3). The model exhibited strong predictive ability (Q^2^ = 0.995) and high explained variance (R^2^Y = 0.999), confirming its stability and reliability for subsequent analysis. Untargeted metabolomics detected a total of 3301 known metabolites across both groups, which were classified into 176 distinct categories. The predominant classes of metabolites were identified as follows: carboxylic acids and derivatives (133 metabolites), fatty acyls (105 metabolites), organooxygen compounds (88 metabolites), benzene and substituted derivatives (86 metabolites), flavonoids (72 metabolites), and prenol lipids (61 metabolites).

The analysis identified a total of 1139 DAMs. Marked metabolic differences were found between the HS and LS varieties. Specifically, 414 metabolites were upregulated and 725 downregulated in HS (Figure 4B). Furthermore, the DAM profiles of the two varieties exhibited marked differences (Figure 4C). The KEGG results showed that the differential metabolites were associated with 66 metabolic pathways (Figure 4D). Enrichment analysis identified the following pathways as most significant: isoflavonoid biosynthesis (map00943), biosynthesis of secondary metabolites (map01110), arginine biosynthesis (map00220), and pentose phosphate pathway (map00030).

### 2.5. Differential Metabolite Analysis

**Figure 5:**
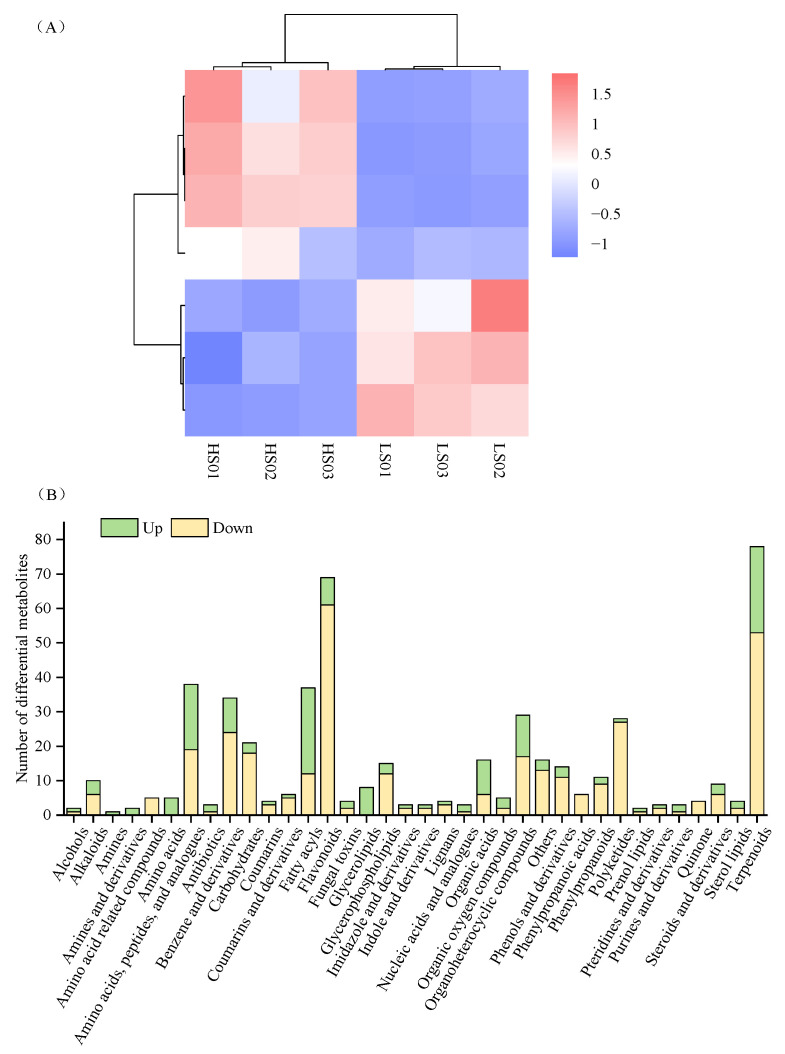
DAM profiling in HS and LS vegetable soybean varieties. (**A**) Heatmap of DAMs associated with sweetness in different samples. (**B**) Histogram of the DAMs category annotated in different vegetable soybean samples.

To investigate the metabolic basis of sweetness variation between HS and LS vegetable soybeans, the differentially accumulated metabolites (DAMs) were functionally categorized. Among the 505 annotated DAMs, the most abundant categories were terpenoids, flavonoids, amino acids (including peptides and analogues), fatty acyls, and benzene derivatives (Figure 5B). Notably, most metabolites in the terpenoid and flavonoid pathways were downregulated in the HS variety. These compounds may have a limited contribution to sweetness perception. In contrast, all amino acid-related DAMs showed significant upregulation in the HS variety, including L-serine. Of particular interest, L-serine possesses intrinsic sweetness and serves as a precursor for glycine and cysteine, potentially enhancing sweetness perception through synergistic effects.

Additionally, approximately 50% of the DAMs in the category of amino acids, peptides, and analogues were found at higher levels in the high-sweetness group; one example is threonine (sweet-tasting). Within the carbohydrate category, most DAMs were downregulated in the HS variety (e.g., D-(-)-ribose, D-(-)-3-phosphoglyceric acid, and 6-O-phosphono-D-gluconic acid), whereas three carbohydrate derivatives exhibited upregulation. Among organic acids, key metabolites such as dibutyl malate were significantly enriched in the HS variety. Furthermore, a key DAM formylkynurenine—an intermediate in the tryptophan-to-alanine pathway—showed upregulation in the HS variety. This upregulation likely promotes the accumulation of the sweet-tasting amino acid alanine in vegetable soybeans. Through screening, a total of 7 DAMs were identified (Figure 5A).

### 2.6. Integrated Analysis of Differentially Expressed Genes and Metabolites Associated with Seed Sweetness

Based on the above results, we identified 7 metabolites and 17 genes involved in multiple metabolic pathways closely associated with sweetness. These 17 DEGs were all related to the regulation of sweetness in vegetable soybeans. Among them, only three (*HXK2*, *INV*, *STS1*) were downregulated in the HS variety, while the remaining 14 were all upregulated. Notably, *FBP* and *PCKA*, which encode the gluconeogenic rate-limiting enzymes fructose-1,6-bisphosphatase and phosphoenolpyruvate carboxykinase, were upregulated, a change expected to promote glucose accumulation and sucrose synthesis. The genes *PFK3*, *HXK2*, and *PKP2* encode key rate-limiting enzymes of glycolysis, whereas *FBA2*, *PGI1*, *PGK1*, and *GAPCP1* encode central enzymes that regulate the glycolytic/gluconeogenic carbon flux. *AK1* encodes an enzyme catalyzing the conversion of aspartic acid, a precursor for threonine biosynthesis. *INV* encodes a pivotal enzyme responsible for sucrose degradation, and *TS1* encodes the enzyme that directly catalyzes threonine synthesis. Conversely, the downregulation of *STS1* likely inhibits stachyose accumulation.

**Figure 6:**
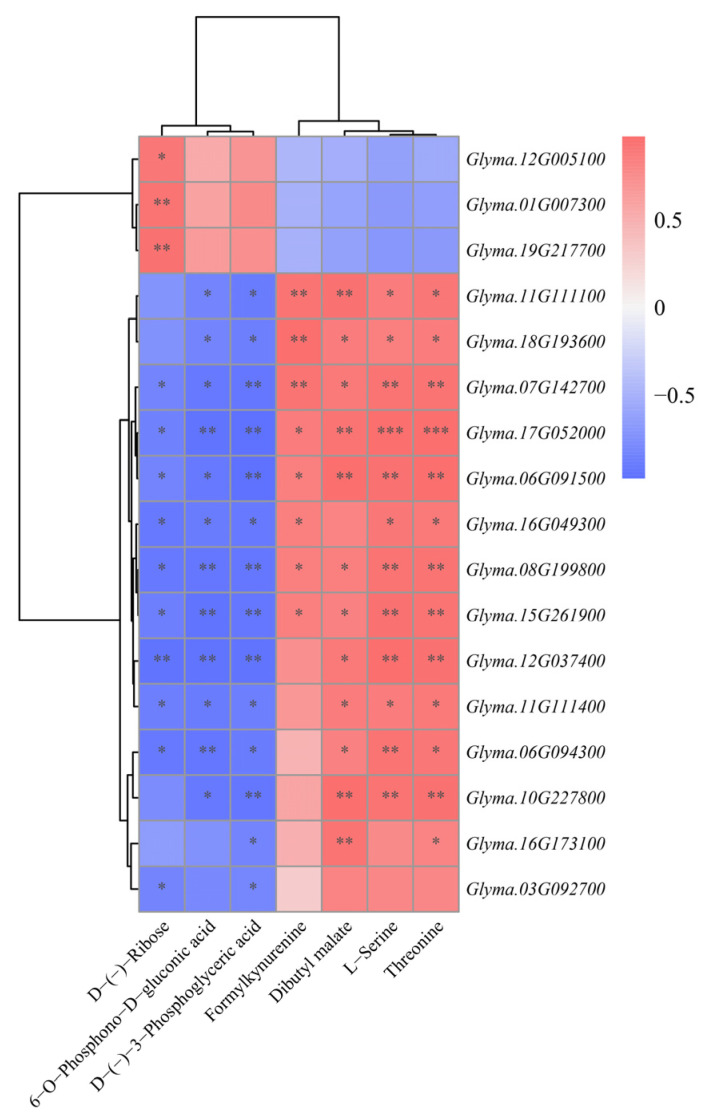
Pearson correlation analysis between sweetness-related genes and metabolites in vegetable soybean. *, **, *** indicate significant difference at *p* < 0.05, *p* < 0.01 and *p* < 0.001, respectively.

To investigate the correlations and interactions between genes and metabolites in regulating the sweetness of vegetable soybeans, a Pearson correlation analysis was conducted (Figure 6). Utilizing rigorous screening criteria, namely an |*r*| > 0.8 and a *p*-value < 0.05 [28], allowed for the identification of substantial correlations between genes and metabolites. Based on this standard, significant associations between genes and metabolites were determined. According to Pearson correlation analysis, the expression of DEGs in the glycolytic/gluconeogenesis pathway was strongly positively correlated with the contents of L-serine and threonine (Figure 6). Formylkynurenine was also positively correlated with multiple DEGs, indicating that enhanced glucose metabolism coincides with the accumulation of sweet-tasting amino acids. The level of dibutyl malate was positively associated with the expression of most DEGs, which is consistent with the higher malic acid content observed in the high-sweetness group. However, D-(-)-Ribose, D-(-)-3-Phosphoglyceric acid, and 6-O-Phosphono-D-gluconic acid showed strong negative correlations with DEGs in the glycolysis/gluconeogenesis pathway. This suggests that the pentose phosphate pathway may be suppressed, thereby favoring gluconeogenesis and sucrose synthesis in the HS variety.

### 2.7. Correlation Between Putative Transcripts and Metabolites Associated with Sweetness Formation

**Figure 7:**
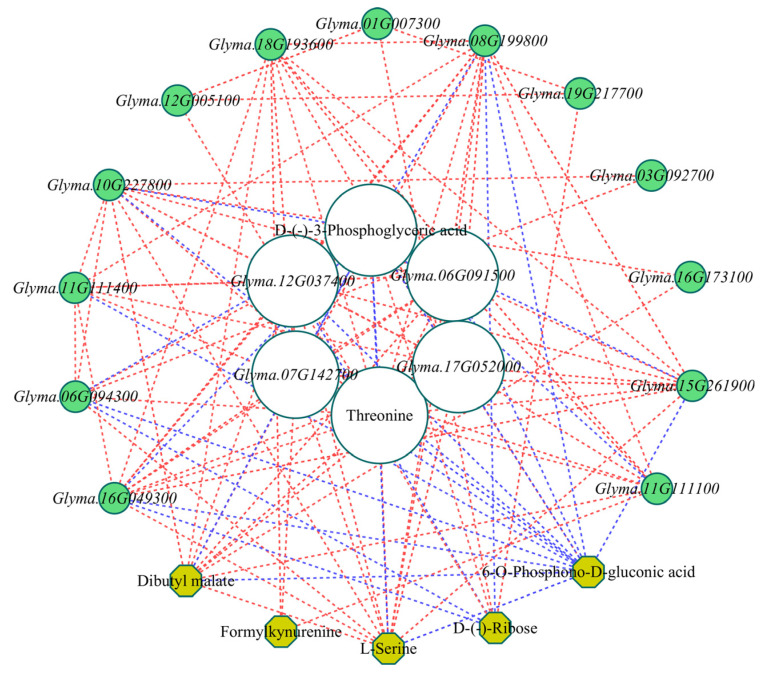
Network analysis of DAMs and DEGs associated with sweetness formation in vegetable soybeans. Yellow nodes represent metabolites, and green nodes represent genes. Line colors denote correlation type (blue: negative; red: positive), and node size reflects connectivity strength.

Pearson correlation was performed between DAMs and DEGs based on metabolite peak areas and transcript FPKM values. A gene–metabolite correlation network was constructed using in Cytoscape (Figure 7). This network included components based on significant correlation thresholds (|*r*| > 0.8, *p* < 0.05). The resulting network comprised 7 metabolites and 17 genes, from which 6 key hub nodes with high connectivity were identified, including two DAMs (Threonine and D-(-)-3-Phosphoglyceric acid) and four DEGs (*FBA2*, *Glyma.12G037400*; *TS1*, *Glyma.17G052000*; *PCKA*, *Glyma.06G091500*; and *FBP*, *Glyma.07G142700*).

Threonine had the highest node connectivity (16), followed by *FBA2* (15). In contrast, *GAPCP1* (*Glyma.03G092700*) was connected to the fewest nodes (2). *FBP* showed significant positive correlations with *AK1* (*Glyma.16G049300*), *FBA2* (*Glyma.12G037400*), *PFK3* (*Glyma.08G199800*), *TS1* (*Glyma.17G052000*), *PCKA* (*Glyma.06G091500*), and *PGK1* (*Glyma.15G261900*), and negative correlations (*p* ≤ 0.01, *r* < −0.9) with D-(-)-3-phosphoglyceric acid and 6-O-phosphono-D-gluconic acid. *PCKA* was significantly positively correlated with *AK1*, *FBA2*, *FBP*, *PFK3*, *TS1*, *PGK1*, *PKP2* (*Glyma.10G227800*), L-serine, threonine, and dibutyl malate, and negatively correlated with 6-O-phosphono-D-gluconic acid and D-(-)-3-phosphoglyceric acid. The high clustering coefficient of *TS1* (0.76) indicates that it plays a central role in the threonine biosynthesis pathway. According to the data, *FBA2*, *TS1*, *PCKA*, and *FBP* are key genes that modulate sweetness development.

### 2.8. Validation of the Transcriptome Data

**Figure 8:**
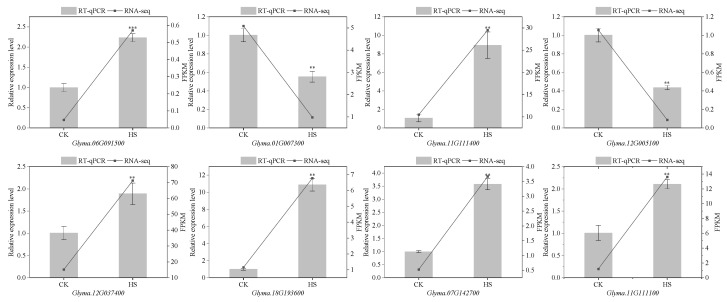
Validation of gene expression patterns derived from transcriptome data. The histograms show the results of RT-qPCR, whereas the line chart presents the result of RNA-seq. Data are presented as mean ± SD (*n* = 3 biological replicates per group). **, *** indicate significant difference at *p* < 0.01 and *p* < 0.001, respectively.

Our results revealed that genes responsible for encoding components involved in glycolysis/gluconeogenesis, starch and sucrose metabolism, and amino acid biosynthesis, were associated with sweetness variation in vegetable soybean. Accordingly, eight genes were selected from these metabolic pathways. Further verification via RT-qPCR analysis demonstrated that the RT-qPCR results were highly consistent with the RNA-seq datasets, thus supporting the reliability of the RNA-seq data (Figure 8).

## 3. Discussion

Fructose-bisphosphate aldolase is a key enzyme in the glycolysis and gluconeogenesis pathways, contributing to glucose metabolism, and participates in carbon fixation as well as sucrose metabolism [29,30,31]. Fructose-1,6-bisphosphatase (FBP) and sucrose-phosphate synthase (SPS) are crucial for sucrose biosynthesis. FBP generates fructose-6-phosphate, which is then used by SPS as the direct substrate to form sucrose-phosphate. Consequently, increased activities of both enzymes synergistically enhance sucrose synthesis [32]. The enzyme invertase (INV) converts sucrose into glucose and fructose [33]. In the present study, three *FBA2* genes (*Glyma.11G111100*, *Glyma.11G111400*, *Glyma.12G037400*) and two *FBP* genes (*Glyma.07G142700*, *Glyma.18G19360*) were identified, all of which were significantly upregulated in the HS variety. The upregulation of *FBA2* promotes the key glycolytic reaction responsible for the reversible transformation of fructose-1,6-bisphosphate into glyceraldehyde-3-phosphate and dihydroxyacetone phosphate. This process facilitates the accumulation of downstream triose phosphates, which can then serve as substrates for gluconeogenesis to be ultimately converted into direct precursors like UDP-glucose for sucrose synthesis. Elevated expression of *FBP* channels carbon flux through gluconeogenesis, hydrolyzing fructose-1,6-bisphosphate (FBP) to fructose-6-phosphate (F6P) and directing it toward the sucrose-phosphate synthase (SPS)-driven sucrose synthesis pathway. Concurrently, this upregulation inhibits the reverse glycolytic reaction, establishing unidirectional metabolic flux control. Specifically, the synergistic upregulation of *FBA2* and *FBP* in the HS variety coordinately directs carbon flux toward sucrose biosynthesis. *FBA2* activity expands the pool of triose phosphates, supplying substrate for gluconeogenesis, while fructose-1,6-bisphosphatase channels this flux by hydrolyzing FBP to F6P, directing carbons toward UDP-glucose and sucrose synthesis. Furthermore, downregulation of *INV* (*Glyma.12G005100*) significantly inhibits sucrose degradation, further promoting net sucrose accumulation. The enzyme phosphoglucose isomerase (PGI), which catalyzes the interconversion of F6P and glucose-6-phosphate (G6P), plays a key role in directing cellular carbon metabolic flux. In addition to this PGI-mediated interconversion, the enzymatic phosphorylation of glucose and fructose by cytosolic hexokinase or fructokinase can likewise generate F6P and G6P [34]. These coordinated metabolic changes ultimately drive the increase in sugar content observed in the HS variety.

Plants absorb inorganic nitrogen from the soil, which is first assimilated into glutamine and glutamate [35]. Notably, asparagine serves as the primary nitrogen transporter due to its high nitrogen-to-carbon ratio (2N:4C) [36]. In this experiment, significant upregulation of gene *AK1* (*Glyma.16G049300*) was observed in the HS variety, which helps maintain redox balance and ensures efficient synthesis of sweet-tasting amino acids. The aspartate produced by *AK1* serves as a precursor for threonine synthesis, thereby promoting threonine accumulation. Concurrently, the upregulation of *TS1* (*Glyma.17G052000*) directly enhances threonine synthesis in the HS variety. This molecular change corresponds to the higher free amino acid content of aspartate and threonine in the HS variety. In addition, the enrichment of other sweet amino acids (glycine, proline, alanine, serine) in the HS variety further strengthened the sweetness perception of vegetable soybeans.

In addition to the pathways described above, certain DEGs could further influence sweet taste via additional metabolic or signaling pathways. The enzyme HXK catalyzes the phosphorylation of glucose in the glycolytic pathway, which is a rate-limiting step of glycolysis [37]. The downregulation of *HXK2* (*Glyma.01G007300*) in the HS variety can reduce glucose consumption. Furthermore, hexokinase proteins catalyze hexose phosphorylation and are important for sugar sensing and signal transduction [38]. The downregulation of *STS1* (*Glyma.19G217700*) in the HS variety may limit stachyose synthesis, consistent with the lower stachyose content observed in the HS variety compared to the LS variety. Typically, organic acids are observed to accumulate to high levels during the early stages of plant development. However, their concentrations gradually decrease with maturation [39]. This reduction is attributed to enhanced sugar synthesis and maturation-related secondary metabolism [40]. In the HS variety, the reduction of citrate and malonate alongside the accumulation of malate indicates a shift in TCA cycle flux towards the malate node. The upregulation of *PKP2* (*Glyma.10G227800*, *Glyma.16G173100*) further drives carbon flux into energy metabolism and sugar synthesis by enhancing the transformation of phosphoenolpyruvate to pyruvate, thereby reducing the retention of organic acid intermediates.

Metabolomic analysis revealed that the downregulation of terpenoids and flavonoids in the high-sweetness vegetable soybean may reflect a trade-off in carbon and energy allocation between primary and secondary metabolism. In plants, the biosynthesis of sugars and secondary metabolites such as terpenoids and flavonoids shares common precursors derived from photosynthesis [41]. The accumulation of sugars in the HS variety likely diverts carbon flux toward primary metabolic pathways (e.g., glycolysis, starch and sucrose metabolism), thereby reducing the precursor supply for secondary metabolism. Previous studies have shown that manipulating primary metabolism can affect fruit quality traits, including the levels of phenolics and terpenoids, and that carbon allocation to secondary metabolites is actively coordinated with other metabolic sinks [42,43]. Regardless of the underlying mechanism, terpenoids and flavonoids are generally bitter or astringent. Their reduced levels may decrease off-flavor compounds, indirectly contributing to the improved sweetness of vegetable soybean. Flavonoids and terpenes are recognized as bitter components in many plant-based foods [44], and non-nutrient bioactive substances in plant-based foods also contribute to astringency and bitterness [45].

To understand whether the sweetness-related changes observed in vegetable soybean also occur in other crops, we compared our results with previously published studies. In sweet corn [46], researchers have systematically dissected the genetic basis of flavor formation and found that flavor complexity goes well beyond a simple “sweet” taste; instead, it is an integrated perception involving sugars, acids, and volatile compounds. Through multi-omics analysis, three new flavor-related genes were identified. *ZmAPS1* regulates adenosine metabolism and affects the synthesis of flavor precursors. *ZmSK1* influences quinic acid synthesis and is closely linked to sweet corn aroma. *ZmCRR5* modulates fructose accumulation and plays a key role in balancing sweetness and yield. In contrast, studies in tomato provide more precise quantitative benchmarks. One study compared vine-ripened (VR) and postharvest-ripened (PR) tomato fruits and found that VR fruits had 29.31%, 32.32%, and 65.45% higher sucrose, fructose, and glucose contents, respectively, than PR fruits [22]. Most DEGs involved in glycolysis/gluconeogenesis, starch and sucrose metabolism were upregulated in VR fruits. In our study, the HS vegetable soybean had approximately 24% higher sucrose, 235% higher fructose, and 28% higher glucose than the LS variety. Meanwhile, 72–78% of DEGs in glycolysis/gluconeogenesis, starch and sucrose metabolism pathways were upregulated in HS, showing a transcriptional pattern similar to that reported in the published study. More importantly, another tomato study used gene editing to knock out Sl*CDPK27* and Sl*CDPK26*, which act as sugar brakes by phosphorylating sucrose synthase SUS3 [47]. This increased glucose and fructose contents by 35% and 30%, respectively, without affecting fruit weight or yield. Broccoli presents a distinct contrast. Chevilly et al. [48] analyzed 20 cultivars and found no significant correlation between sugar content and taste score (R^2^ < 0.1). Instead, GABA correlated positively with taste, while leucine, lysine, alanine and myo-inositol showed negative correlations. In vegetable soybean, sucrose content was positively correlated with sweetness, and sweet-tasting amino acids (serine, alanine, glycine, proline) accumulated to higher levels in the HS variety. This comparison shows that the metabolic basis of sweetness is species-specific: sugars are not the main determinant of taste in broccoli, whereas both sugars and certain amino acids contribute to sweet taste in vegetable soybean. Collectively, these cross-species comparisons indicate that the high-sweetness trait in vegetable soybean is closely associated with transcriptional upregulation of sugar metabolism pathway genes, a regulatory pattern similar to those reported in tomato and sweet corn, but distinct from broccoli where sugars are not the main drivers of sweetness. Therefore, sweetness formation in vegetable soybean is primarily driven by sucrose accumulation and coordinated activation of related metabolic pathways.

In conclusion, we propose that the enhanced sweetness of high-sweetness vegetable soybean results from two complementary aspects. On the one hand, direct positive factors include the upregulation of sucrose biosynthesis-related genes (such as *FBA2* and *FBP*), which promote sucrose accumulation, along with increased levels of sweet-tasting amino acids (serine, alanine, glycine, proline). On the other hand, indirect contributing factors involve the reduced accumulation of bitter or astringent compounds (e.g., terpenoids and flavonoids) in the high-sweetness variety. This weakens off flavor interference and makes the sweetness more perceptible. Accordingly, the superior sweetness of vegetable soybean likely arises from the combined effects of increased sugars and sweet amino acids, as well as reduced bitter compounds.

## 4. Materials and Methods

### 4.1. Materials

In total, 287 soybean germplasm accessions from different regions in China were collected and selected as experimental materials for this study. The sweetness of the samples was measured using an electronic tongue (ASTREE, Alpha M.O.S company, Toulouse, France). Based on the results (Table S1 & Figure S1 in the Supplementary Material), the varieties with the highest and lowest sweetness were selected for further investigation. The selected soybean varieties were cultivated under identical environmental conditions at the research base of Liuhe (Nanjing, China), and the study employed a triplicate design. Each plot consisted of seven rows, each measuring four meters in length, with a spacing of 0.4 m between rows and 0.15 m within rows. Soybeans were harvested from the central five rows, while the border rows served as isolation buffers. For subsequent multi-omics analysis, seeds of each sample that were healthy and undamaged were collected at the R6 stage.

### 4.2. Analysis of Soluble Sugar, Free Amino Acid and Organic Acid Composition

Soluble sugar components were measured following a previously described method with an Agilent 1260 HPLC system (Agilent Technologies, Santa Clara, CA, USA) (Chinese National Standard GB 5009.8-2023) [49]. The freeze-dried sample was extracted with water containing zinc acetate and potassium ferrocyanide solutions. After centrifugation, the centrifuged sample was then subjected to filtration through a 0.45 μm syringe filter, with the filtrate collected in a 1.5 mL vial for subsequent HPLC-RID analysis.

Free amino acids were extracted, derivatized pre-column with o-phthalaldehyde (OPA), and then separated and quantified by HPLC (Agilent 1100, Agilent Technologies, Santa Clara, CA, USA). A standard mixture of twenty amino acids was used for quantitative analysis.

Organic acids were extracted according to a protocol reported by Chinese National Standard GB 5009.157-2016 [50]. Extraction was performed using metaphosphoric acid solution in an ice-water bath.

### 4.3. Metabolite Extraction and Analysis

High-sweetness and low-sweetness vegetable soybean samples were used in this study, with six biological replicates per group. Sample powder (50 mg) was thoroughly mixed with a methanol–water solution. The mixture was first homogenized at 50 Hz for 10 min using a tissue homogenizer, then ultrasonicated in an ice-water bath for 30 min. For quality control, 20 µL of each solution was filtered and combined to create a composite quality control (QC) sample. This sample was then utilized to assess the consistency and reliability of the LC/MS analysis. Both the individual filtrates and the pooled QC sample were then placed in vials for LC/MS analysis. The acquisition of mass spectrometric data on the Q Exactive HF instrument was accomplished through a DDA mode, acquiring both full-scan MS (*m*/*z* 100–1500) and MS/MS spectra, employing negative and positive ionization methods. For MS/MS acquisition, the top 3 most intense precursor ions (based on intensity) were sequentially isolated.

### 4.4. MS Data Analysis

The data output from the Compound Discoverer program was integrated and processed within metaX (version 2.0.0) [51], facilitating subsequent investigation and analysis. Cluster analysis was performed to examine metabolite accumulation patterns across sample groups. The variable importance in projection (VIP) from the orthogonal partial least squares discriminant analysis (OPLS-DA) model and the fold change (FC) from univariate analysis were used to identify differential metabolites, with thresholds of VIP ≥ 1 and FC ≥ 1.5 or FC ≤ 0.5 (q-value < 0.05). Pathway enrichment analysis was conducted based on the Kyoto Encyclopedia of Genes and Genomes (KEGG) database, with a false discovery rate (FDR) threshold of 0.05 after Benjamini–Hochberg correction. The organism used for the analysis was soybean (*Glycine max*).

### 4.5. RNA Extraction and Transcriptome Analysis

RNA extraction was performed with the TRIzol reagent kit (Invitrogen, Carlsbad, CA, USA) in strict accordance with the supplier’s instructions. This purified mRNA was chemically fragmented and then used to synthesize first-strand cDNA. The double-stranded cDNA generated was then subjected to a series of processing steps, including end repair, 3′ ends A-tailing, and ligation with Illumina sequencing adapters (Illumina Novaseq X Plus platform, Illumina, San Diego, CA, USA). The adapter-ligated products were purified using AMPure XP Beads (1.0×) and then amplified by PCR. The final constructed cDNA libraries were used for sequencing. High-quality clean reads were obtained by processing the raw sequencing data with fastp, which performed adapter trimming, removal of reads containing more than 10% ambiguous bases (N), and quality filtering (reads were discarded if more than 50% of bases had a quality score ≤ 20). For each sample, transcript assemblies were generated from the reads mapped to the soybean reference genome (*Glycine max* Wm82.a4.v1) using StringTie (v1.3.3b). To assess overall sample relationships, PCA was conducted utilizing the gmodels package (version 2.19.0) in R (version 4.4.1), and the results were visualized using the ggplot2 package (version 4.0.2). Differential expression analysis was conducted using the DESeq2 package (version 1.48.0) [52], with thresholds of |log2FC| > 1 and FDR < 0.05 for defining significantly differentially expressed genes. Volcano plots and enrichment result visualizations were generated using the ggplot2 package, while heatmaps were created with the pheatmap package (version 1.0.13) after z-score normalization and hierarchical clustering. GO and KEGG enrichment analyses were performed using the hypergeometric test, with an FDR ≤ 0.05 considered significant.

### 4.6. Quantitative Real-Time Polymerase Chain Reaction (RT-qPCR) Assay

High-sweetness (HS) and low-sweetness (LS) vegetable soybean samples were used, with three biological replicates per group. Total RNA was extracted using the RNAprep Pure Plant Kit (DP432, Tiangen, Beijing, China). Gene-specific primers were designed with SnapGene (Table S2), with *actin* serving as the internal reference control. Quantitative real-time PCR was performed using ChamQ Blue Universal SYBR qPCR Master Mix (Vazyme, Nanjing, China) on a QuantStudio5 Real-Time PCR System (Thermo, Shanghai, China). The RT-qPCR reaction mixture (20 μL total volume) contained 10 μL of ChamQ Blue SYBR Master Mix (Vazyme, Nanjing, China), 0.4 μL each of forward and reverse primers, 2 μL of cDNA template, and 7.2 μL of ddH_2_O. The thermal cycling program was as follows: initial denaturation at 95 °C for 30 s, followed by 40 cycles of 95 °C for 10 s, 60 °C for 15 s, and 72 °C for 20 s. Fluorescence signals were acquired at the end of each cycle. All reactions were performed in triplicate. Relative expression was calculated using the 2^−ΔΔCT^ method. Eight candidate genes were selected from the identified DEGs involved in glycolysis/gluconeogenesis, starch and sucrose metabolism, and amino acid biosynthesis pathways (*Glyma.06G091500*, *Glyma.11G111100*, *Glyma.11G111400*, *Glyma.12G037400*, *Glyma.07G142700* and *Glyma.18G19360* upregulated in HS; *Glyma.01G007300* and *Glyma.12G005100* downregulated in HS).

### 4.7. Statistical Analysis

Biological replicates were as follows: three per variety for transcriptomics, RT-qPCR, and chemical composition assays; six per variety for metabolomics. All measurements were performed in at least triplicate. Data analysis was performed using Microsoft Excel 2010, and graphs were generated with Origin 2021 (OriginLab Corporation, Northampton, MA, USA). Significant differences were detected by one-way ANOVA using SPSS v26.0 (SPSS Inc., Chicago, IL, USA). The following analyses were performed using R software (version 4.4.1): principal component analysis (PCA), volcano plots, and bubble charts were generated using the ggplot2 package; differential expression analysis was conducted using the DESeq2 package; for the correlation heatmap between genes and metabolites, correlation coefficients and *p*-values were calculated using the Hmisc package (version 5.2-5), and the heatmap was created using the pheatmap package (version 1.0.13); for network analysis, the correlation matrix was calculated using the WGCNA package (version 1.74) to generate edge lists, and the network was visualized using Cytoscape (version 3.8.2).

## 5. Conclusions

Chemical analysis revealed that the HS variety contains significantly greater amounts of soluble sugars (particularly sucrose) and sweet-tasting amino acids, such as serine, alanine, glycine, and proline. To investigate this, transcriptomic and metabolomic analyses were conducted. Integrated data revealed that genes and metabolites related to starch and sucrose metabolism, glycolysis/gluconeogenesis, and amino acid biosynthesis pathways play critical roles in sweetness regulation. Furthermore, genes including *PCKA* (*Glyma.06G091500*), *HXK2* (*Glyma.01G007300*), *INV* (*Glyma.12G005100*), three *FBA2* (*Glyma.11G111100*, *Glyma.11G111400*, *Glyma.12G037400*), and two *FBP* (*Glyma.07G142700*, *Glyma.18G19360*) were identified as key regulatory factors determining sweetness levels. These results elucidate the molecular basis of sweet flavor formation in vegetable soybean seeds, providing a foundation for future molecular studies and the breeding of high-quality vegetable soybean varieties.

## Data Availability

Data will be made available on request.

## Supplementary Materials

The following supporting information can be downloaded at: https://www.mdpi.com/article/10.3390/molecules31091485/s1, Figure S1: Frequency distribution of the sweetness response values of 287 vegetable soybean resources; Table S1: Phenotypic variation for the sweetness response values; Figure S2: GO enrichment analysis of DEGs identified in high-sweetness (HS) and low-sweetness (LS) vegetable soybeans; Table S2: List of primers used for RT-qPCR analysis of candidate gene expression; Figure S3: OPLS-DA score plot of the HS and LS varieties. Data S1. RT-qPCR gene expression levels.

## Abbreviations

The following abbreviations are used in this manuscript:

Ala	Alanine
DEGs	Differentially Expressed Genes
FDR	False Discovery Rate
Gly	Glycine
GO	Gene Ontology
HS	High-sweetness vegetable soybean
KEGG	Kyoto Encyclopedia of Genes and Genomes
LS	Low-sweetness vegetable soybean
PCA	Principal Component Analysis
Pro	Proline
Ser	Serine
DAMs	Differentially accumulated metabolites
FBP	Fructose-1,6-bisphosphatase
FBA2	Fructose-bisphosphate aldolase
HXK	Hexokinase
