# Supplementary material for: Integrated Transcriptome and Metabolome Analysis Reveals the Mechanism of Sweetness Formation in Vegetable Soybean Seeds
Source: Molecules. 2026 Apr 29;31(9):1485. doi: 10.3390/molecules31091485 (PMC13164844; doi:10.3390/molecules31091485)
Supplement: Supplementary file 1 [file molecules-31-01485-s001.zip › molecules-4263477-supplementary.pdf]

Supplementary Materials

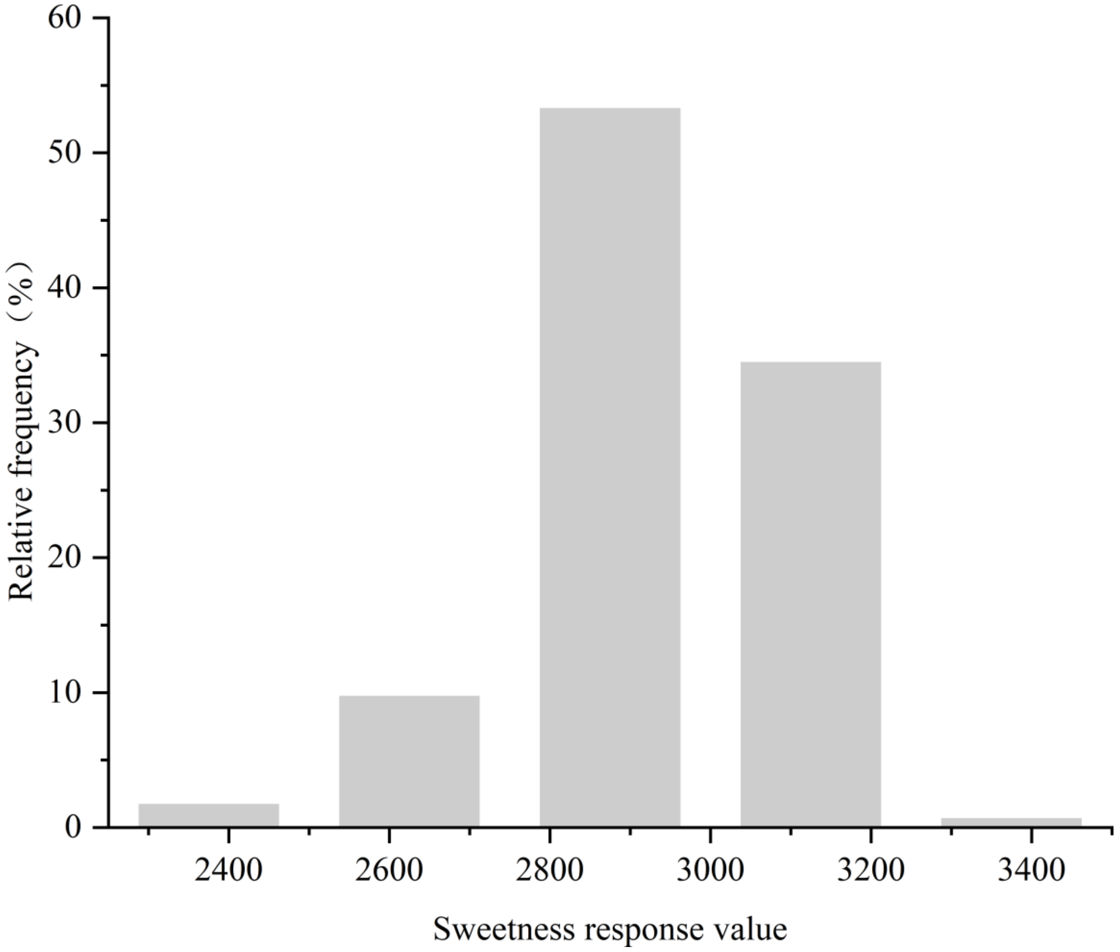

Figure S1. Frequency distribution of the sweetness response values of 287 vegetable soybean resources.

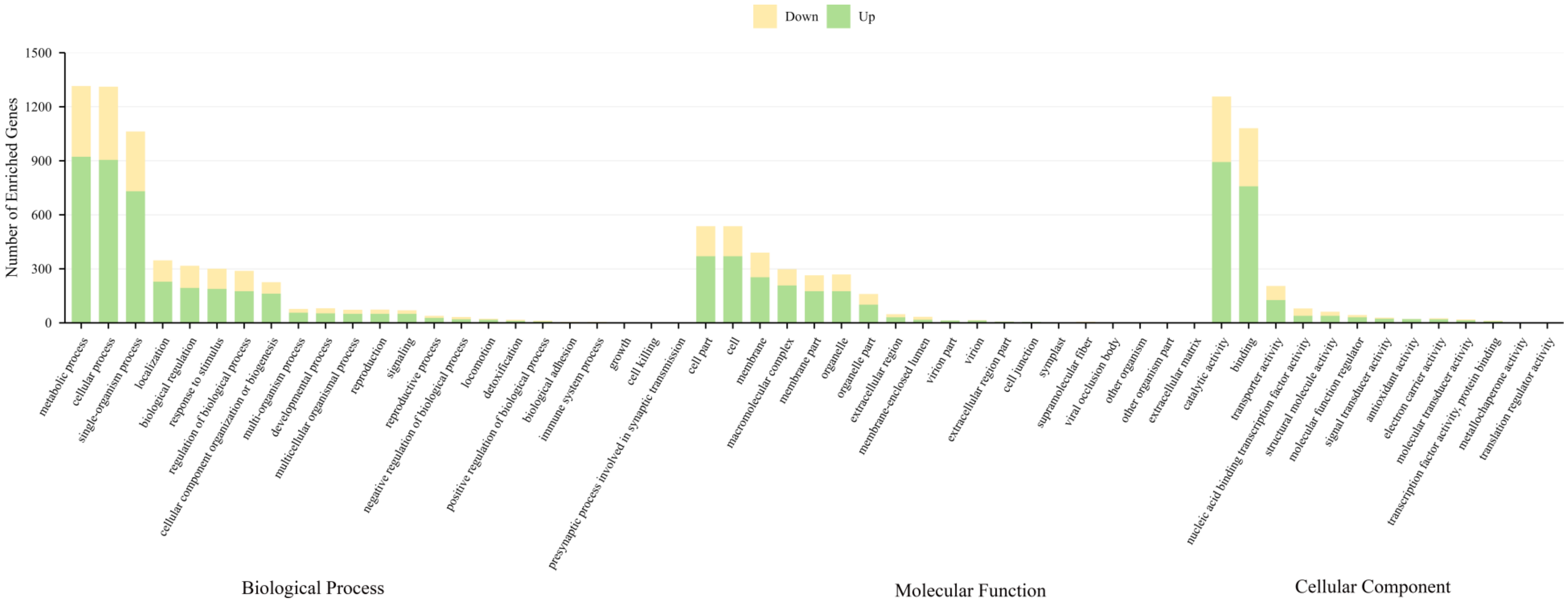

Figure S2. GO enrichment analysis of DEGs identified in high-sweetness (HS) and low-sweetness (LS) vegetable soybeans.

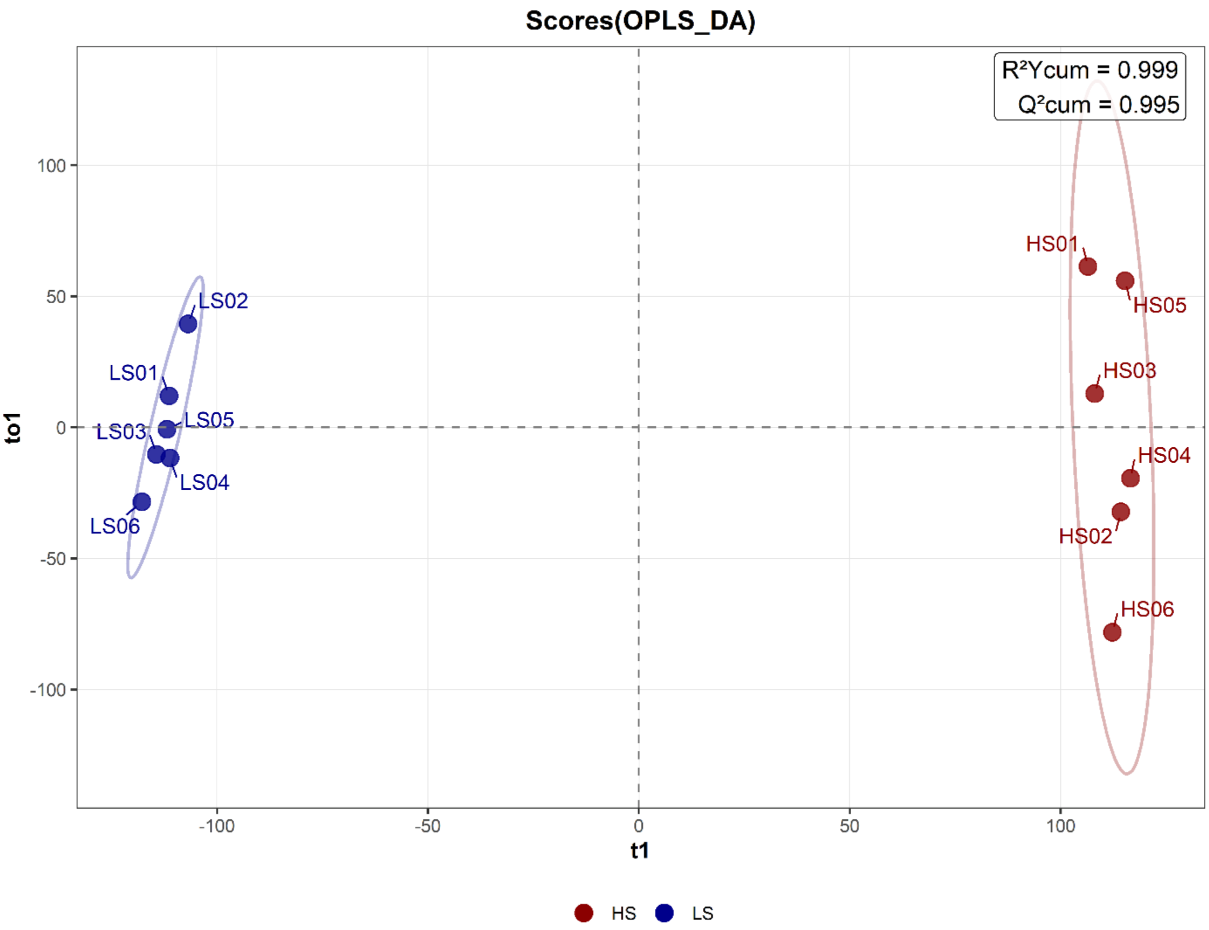

Figure S3. OPLS-DA score plot of the HS and LS varieties.

Table S1 Phenotypic variation for the sweetness response values

Trait	Number	Minimum	Maximum	Mean	SD	CV(%)
Sweetness	287	2300.21	3282.98	2937.53	155.36	5.29

Table S2 List of primers used for RT-qPCR analysis of candidate gene expression

Gene	Forward	Reverse
Glyma.01G007300	GTGGGAAGGAGAAAGGTGTTG	AAACCCAGTTCCCTTTGTCTG
Glyma.11G111100	ATGTTAGGCAGCAGCAACAC	TCAAGTACACAACTCACAGCA
Glyma.11G111400	GGTGCATGGAATACATTTGTC	TGGATGATGATAACTTCAGCC
Glyma.12G037400	CTCATACAAAGGTTGCTGTAG	ATCTCACAACTGATGCAGAAG
Glyma.07G142700	ACGAAGAAGATAAGTGTGGGAC	GAGAAAATCAACTGGGTGGATG
Glyma.18G193600	AGAAGCTGCAACCACTGAGAC	TGAGGTTGGAAATGTTGGCTC
Glyma.12G005100	AGGATCACCATTGGCTCAAAG	AGCCTTCAACACATGTTTCAC
Glyma.06G091500	ATCCGACCCACTCCTAAAGAG	TCATTTCCCCAGCATATTGTG
